# Book Reviews

**Published:** 1877-02

**Authors:** 


### Book Reviews.

[NOTE.—All works reviewed in the pages of the CHICAGO MEDICAL JOURNAL AND EXAMINER may be found in the extensive stock of W. B. KEEN, COOKE & CO., whose Catalogue of Medical Books will be sent to any address upon request.]

DISEASES OF THE NOSE AND ITS ACCESSORY CAVITIES. By *W. Spencer Watson, F.R.C.S., Eng., B.M. Lond., etc.* London: H. K. Lewis.

This book originated in the hope, says the author, "that by condensing and systematizing the vast mass of material" relating to affections of the nose, which is scattered through the literature of medicine, a certain void might be filled up, and the busy practitioner afforded a real help. He is right in this that in the systematic works on general surgery and medicine the disorders of the nose receive but slight attention. Such a book as the one before us is very much needed by the profession. In many ways the work is an excellent one. The author has freely drawn upon a great array of literature upon the subject, and his work doubtless is a fair presentation of much that is best in this literature. But we think the average doctor will be disappointed in the book, from the brevity of those sections devoted to some of the commoner forms of disorders with which he is often perplexed, and the fullness with which those affections are treated of which he very seldom even sees. At the same time some of the treatment recommended for the more common affections will meet with criticism.

The free way in which the author recommends the use of the nasal douche will not be allowed to pass unchallenged by our friends the otologists, nearly all of whom, we notice, look upon a nasal douche with only less terror than they would upon a small infernal machine.

Nevertheless, Mr. Watson's book is a good one. It contains more and better information on the subjects of which it treats than any other we know of; and to any practitioner who cares to be thorough in this department we cheerfully commend the work.

Contributions to Reparative Surgery. By *Gurdon Buck, M.D.* New York: D. Appleton & Co. 1876.

The "Reparative Surgery" of the author embraces a series of operations which he performed in the region of the face, the neck and the upper extremities, to repair the damage sustained by the loss of parts or to remove congenital malformations from excess or arrest of development, or to remedy the cicatricial contractions following burns.

The report of twenty-nine cases (some of which had previously been published in medical periodicals) occupies the greater part of the volume. The accurate description of each case; the conscientious account of every step of the operations, which in several cases numbered five and more; and a collection of good illustrations, showing in each case the original deformity and the permanent result after the lapse of long intervals of time—such features make the reading of the book very interesting and instructive. It is not within the scope of this notice to give even a brief synopsis of the cases comprising: 1, a number of hare-lips and cicatricial contractions involving the eyelids, the face, the chin, the hands and the axilla; 2, a large assortment of reconstructions of the mouth and the nose.

The general observations which precede the report of the cases fairly represent the author's views of plastic operations. In the choice of material which is to supply an existing defect, the author justly warns not to include any cicatricial tissues in a flap, because the same if transplanted will inevitably slough. The truth of this remark is fully substantiated by Case XXVII., in which the author, deviating from his own rule, employed cicatricial skin to cover a large defect; and sure enough, after forty-eight hours sloughing had destroyed all the flaps.

In order to secure a good fit, dressmakers cut the several pieces for a dress after patterns pinned on to the cloth. Dr. B. adopts this method for plastic patch-work: "An exact pattern of the space should be cut from oiled silk, and the pattern applied to the surface which is to supply the new material. Small pins may then be temporarily inserted erect in the skin at intervals around the pattern, and at a distance of one line from its margin, as an allowance for shrinkage."

For the transfer of a portion of skin to a new locality, the author has adopted the usual three methods, viz.: by approximation, by sliding, and by transfer to a distance.

Three kinds of sutures have been employed by the author: The "interrupted thread suture," the "pin suture," and the "beaded wire clamp suture." The latter is intended as an auxiliary for the support of other sutures where it is important to relieve them of strain, and thereby increase the chances of obtaining primary union. They are inserted at a distance of one inch or more from the edges. The adjustment of the edges to each other is achieved by the interrupted sutures, which "should be inserted as closely together as may be necessary to secure exact coaptation of the edges. Their multiplicity is not objectionable, inasmuch as at the expiration of twenty-four hours, when agglutination of the edges will have taken place, their number may be diminished by removing the alternate ones."

To those who yet look upon the material used for sutures as the principal condition for a successful union, we would recommend the following remarks of the experienced writer: "Metallic sutures, in the judgment of the author, possess no advantage over thread, and are as liable to cause ulceration as thread. Both will remain harmless as long as they are needed, provided the edges of the wound they are designed to hold in contact have previously been liberated, so as to be relieved of all strain upon them."

For the insertion of the pin suture Dr. B. has devised and described a "suture pin conductor." We hope the doctor does not love his invention so fondly as to feel aggrieved if we are free to say that the pin suture, in our judgment, is an unnecessary and cruel torture to the patient, and should be thrown aside by modern surgeons. There is no more need for pins in uniting the edges of a hare-lip than in adapting the edges of the eyelids. The modern ophthalmic surgeons employ the interrupted suture only, while Diefenbach yet thought the pin suture was indispensable in tarsorraphy. And from our own experience we are satisfied that the pins can just as safely be dispensed with in the hare-lip sutures.

In regard to the operation for hare-lips, there is another point in which we differ with the author. He exclusively employed Malgaigne's method of paring and uniting the edges of the cleft, asserting that by this method "the formation of a notch at the point of the lip border where the two halves unite is best prevented." Do the author's own results, as represented by the drawings, sustain this assertion? Four of the seven cases show a decided shortening of the lip at the point where the borders of the cleft were united. Especially the results of the operation for double hare-lip, as depicted in Fig. 46 and 56, do not speak well for Malgaigne's method. We think Mirault's operation in such cases gives far better satisfaction; it prevents more efficiently the formation of a notch, and gives greater uniformity to the height of the lip.

To regulate the process of cicatrization of a large granulating surface (as the result of the excision of cicatricial folds from burns), to repress the exuberant granulations, the author entirely relied on the frequent application of solid nitrate of silver and caustic potassa, aided by the compression with adhesive plaster, cut into strips, and applied in immediate contact with the entire granulating surface. It seems he did not employ skin-grafting, which, just for such cases, would have been a valuable auxiliary.

It would have been very interesting to learn the author's views of the value of Reverdin's grafting, as well as of Lister's antiseptic dressing. He did not use the latter in the cases reported; but perhaps he will try it now, since Lister has been in this country to demonstrate the accurate mode of his dressing.

The illustrations in the book are very well executed, the artist having taken great pains to give an accurate copy of the photographs; they are highly interesting as truthful representations of the original deformity of each case and of the final result achieved by the reparative surgery.

The book will be read with a great deal of interest, and its pages will be frequently consulted by those who wish to receive some intelligent information in regard to plastic surgery.

Cyclopædia of the Practice of Medicine, edited by *Dr. H. von Ziemssen*, Professor of Clinical Medicine in Munich, Bavaria. Vol. XI. Diseases of the Peripheral Cerebro-Spinal Nerves, by Prof. Wilhelm Heinrich Erb, of Heidelberg, Baden. Translated by Mr. Henry Power, of London, England. Albert H. Buck, M.D., New York, editor of American edition. 8vo. pp. 623. New York: Wm. Wood & Co. 1876. For sale (by subscription only) by W. T. Keener, 93 Dearborn St., Chicago.

We have read this volume with great interest and much profit. It should be in the library of every physician. It discusses, in quite an exhaustive manner, several diseases that are of almost daily occurrence, and but little understood. It is particularly rich in etiology and pathology, but the bibliography, while it shows the author's great familiarity with the writings of his own country, displays a rather surprising want of acquaintance with those of this country, England and France. The symptomatology is very clear and generally quite exhaustive, and the treatment is fully up to the present advanced position of neurotherapy.

The author's career, like his book, has been a great success; he received his degree as doctor of medicine, in Munich, at the age of twenty-two; at twenty-five he was installed as private instructor in Heidelberg, and first assistant of Friedreich's clinic, and at twenty-eight was made extraordinary professor. He published, prior to the book now before us, twenty important scientific papers.

He preserves the long established division of functional and anatomical diseases of the peripheral nerves, and under the former includes neuralgia, anæsthesia, neuroses of the nerves of special sense, spasm and paralysis; and under the latter hyperæmia, inflammation, atrophy and hypertrophy.

The neuralgias are discussed ably and exhaustively; 173 pages being thus occupied. While we fully endorse all he has written on the etiology of neuralgia, we do not think he has in this, or in the discussion of this important consideration in any of the neuroses, dwelt sufficiently upon immoderate and unnatural sexual indulgence as a powerful cause in the production of this as well as many of the other neuroses of the central as well as the peripheral system. It is our opinion that where the neuropathic predisposition exists there is no one cause that acts so forcibly in the production of these diseases.

Our author writes of the *painful points* of neuralgia, that the frequency of their occurrence has been considerably overestimated by Valleix. We are pleased to have him remark, as confirmation of our own observations, that "in a considerable number of cases it was impossible, even with the most conscientious care, to demonstrate their existence." While discussing the disturbances of nutrition in neuralgia, our author takes occasion to remark that a whole series of the above mentioned trophic disturbances are only explicable on the supposition that the nerves (trophic) exert a direct influence upon the nutrition of the tissues, "thus placing himself on record as a believer in the existence of special trophic nerves."

The articles on neuralgia of the fifth nerve is an excellent one. He dwells upon heredity as the foremost predisposing cause; its greater frequency in middle life and old age and in women, the effect of sexual excess, overstimulation, anæmia and general disturbance of nutrition, the advent of senescence with fatty degeneration of the tissues and diseases of the arteries, on its development. He notices the occasional influence of malarial infection, wounds, anatomical changes in the nerves and in the brain, irritation of the peripheral nerves and excessive psychical activity in its production. The profession generally will endorse his statement that "the treatment of prosopalgia only too frequently presents extraordinary difficulties, taxing the knowledge and patience of the physician to the utmost, and in spite of all efforts giving an unsatisfactory result."

The article on paralysis in general, and the special paralysis of the facial nerve is thoroughly scientific and will well repay careful perusal. Attention is called to the three lesions that may characterize paralysis, as follows: 1st, Destruction or functional incapacity of the motor central apparatus. 2d, Diminution or abolition of the conductivity of the motor nerves; and, 3d, Abolition of excitability and contractility of the muscle.

The different degrees of vaso-motor and associated disturbances occurring in peripheral, spinal and cerebral paralysis are thus described and explained: "Paralysis of the vaso-motor nerves is of frequent occurrence, and first leads to hyperæmia with slight œdema and subsequently to general atony of the vessels, cyanosis and defective nutrition. These symptoms may be chiefly expected in those cases where the vaso-motor nerves are coincidently affected by the primary cause of the paralysis and they are almost certain to occur in cases ot paralysis of peripheral nerves, where the vaso-motor nerves run in the same trunk. They are less frequent in paralysis originating in the spinal cord, because the two sets of fibres are then probably more or less widely separated. Lastly, they are seldom seen in cerebral paralysis, in which the vaso-motor nerves often remain quite intact, since in the brain the vaso-motors run more or less completely detached from the motor nerves."

Our author devotes considerable space to a consideration of the electrical examination of the paralyzed nerves and muscles, and dwells upon its importance in the pathology, and in the differential and local diagnosis. This important subject is very satisfactorily elucidated. Under general treatment he states the truism "that the first and most important point is the determination and fulfillment of the indicated causalis."

In the article on facial paralysis there is quite an exhaustive dissertation on the importance and method of diagnosing the immediate cause and exact seat of the lesion. In the same article the indications of treatment are clearly stated, and in this, as in the treatment of functional neuroses generally, electricity is given the prominent position to which clinical experience entitles it.

Space will not permit a detailed review of the "Anatomical Diseases of the Peripheral Nerves." We find here, however, the same clearness and thoroughness that characterizes the book generally. D. R. B.

CYCLOPÆDIA OF THE PRACTICE OF MEDICINE. Edited by *Dr. H. von Ziemssen*, Professor, etc. Volume IV. Diseases of the Respiratory Organs. By Dr. Fraenkel, of Berlin; Prof. von Ziemssen, of Munich; Prof. Steiner, of Prague; Dr. Riegel, of Cologne; and Dr. Fraentzel, of Berlin. Translated by J. B. Yeo, M.D., of London; J. S. Cohen, M.D., of Philadelphia; A. B. Ball, M.D., and G. M. Lefferts, M.D., of New York; and E. W. Schauffler, M.D., of Kansas City. Albert H. Buck, M.D., of New York, Editor of American Edition. New York: Wm. Wood & Co. 1876. For sale (by subscription only) by W. T. Keener, 93 Dearborn street, Chicago.

The character of this cyclopædia is already familiar to the profession, and this volume does not differ from those which have preceded it in general arrangement and in the excellence of its print. It is divided into five sections, which are contributed by as many authors.

As a rule the subjects have been thoroughly treated; yet in some cases, the authors have almost ignored the opinions of eminent men, who differ from them, while each has elaborated at great length his own pet theory. This is hardly fair, if the work is intended to give us *all* that is known upon the subjects of which it treats; and if it is designed only as a dictionary of facts, as its name implies, it seems much too voluminous, for we are compelled to read page after page to find the facts, which might as well be told in a single paragraph; indeed, prolixity is a prominent fault in this volume. For instance, it takes six pages to inform us how to depress the tongue, and this is a fair illustration of the tediousness of many parts of the book. Aside from these objections and an occasional labored paragraph, we can heartily commend this volume to the profession.

Fraenkel tells us, first, how to inspect the pharynx. He next gives us the history of laryngoscopy, and then describes at length the apparatus needed. Of various illuminating apparatus mentioned, he prefers for ordinary use a concave mirror, which can be attached to the head. The mirror should have a focus of six or seven inches, if we are obliged to use artificial light; but if sunlight (which he considers most desirable) can be employed, the mirror should have a focus of nine or ten inches.

After detailing the method of performing laryngoscopy, he describes the anatomy of the larynx and its image as seen in the laryngeal mirror, at the same time calling attention to the special parts to be observed in an examination, and the visible changes which may be produced in them by disease. An article on rhinoscopy follows; it is succeeded by an interesting chapter on general therapeutics in diseases of the nose, pharynx and larynx. Eighty-two pages by the same author are devoted to diseases of the nose — stenosis and atresia, coryza, purulent catarrh, chronic nasal catarrh, and epistaxis; abscesses and ulcerations, tumors and foreign bodies, and concretions and parasites of the nasal cavity.

Coryza is said to be contagious, and a not infrequent cause of facial erysipelas.

A fair idea of this chapter may be obtained from the article on nose bleed, which occupies eighteen pages. Among the causes of this affection he mentions the hæmorrhagic diathesis and increased lateral pressure in the blood vessels within the nose, induced by hindrance to the flow of blood through the veins, as in the paroxysms of whooping-cough, or by increased arterial pressure, as in hypertrophy of the heart with contraction of the kidneys.

Various infectious diseases, as measles, variola, typhus, etc., and also diseases of the spleen, may give rise to this affection. Epistaxis may become, so to speak, habitual. "It frequently appears vicariously for other hæmorrhages, especially those of a hæmorrhoidal character, or in place of menstruation." But we are cautioned about assigning a vicarious character to hæmorrhages. Habitual nose-bleed is hereditary in some families. The author says: "It is but seldom that fainting, prolonged anæmia, or even death, follows a single hæmorrhage of uncommon severity or a prolonged flow." He does not concur in the opinion held by some authors, that habitual epistaxis during youth induces pulmonary phthisis.

The treatment recommended is, first, pressure of the ala of the affected side against the septum in such a manner as to close the nostril and front part of the nose. Pressure may be made by the fingers introduced into the nostrils, or the anterior tampon may be used. When the bleeding takes place from the anterior part of the nose, as is usually the case, these means will be effectual. As an adjunct the carotid may be compressed. He has seldom seen any good to result from the use of astringent drugs during the attack; but hæmostatics may help to prevent its recurrence. In severe cases the posterior tampon must be employed.

Anæmia, hyperæmia, abnormal color and catarrhal inflammations of the larynx are treated of by von Ziemssen in forty pages.

He states that chronic laryngeal catarrh generally proceeds from an acute attack, as the result of neglect or exposure to such irritating causes as loud talking or singing. Another extremely frequent cause of this affection is chronic pharyngitis, which is especially apt to extend to the larynx when several causes co-operate, such as straining the voice and the abuse of tobacco or alcoholics.

Evidently he does not agree with those who attribute this affection in the majority of cases to phthisis.

Diseases of the trachea and bronchi are treated of by Riegel. He devotes one hundred and forty pages to bronchial catarrh, thirty-two to croupous bronchitis, thirty to stenosis of the trachea and bronchi, twenty to foreign bodies in the trachea and bronchi, and over sixty to bronchial asthma. This article is interesting, but very diffuse.

Nearly two hundred pages are devoted to diseases of the pleura by Fraentzel. We were specially interested in his explanation of the cause of the tympanitic resonance which is commonly obtained over the apex of the pleural cavity when it is nearly filled with fluid. He thinks that the pitch of the sound heard on percussion over the lungs depends not only on the volume of air which they contain, but also on the tension of the pulmonary parenchyma. He states, with Winterich, that as the tension of the parenchyma increases the pitch becomes higher; that as it diminishes the pitch becomes lower; and that with diminution in volume of the lung the pitch becomes higher. As every pleuritic effusion compresses the lung somewhat, its volume becomes less, giving rise to *higher pitch;* but its tension also becomes less, which tends to *lower the pitch.* Therefore, if we accept the above theory, it is at once evident that if the diminution in the volume (*high pitch*) and the diminution in the tension (*low pitch*) are equally balanced, the percussion note under the clavicle will remain unaltered; but if this, as has been stated, is usually deep (*tympanitic*) in moderately large pleuritic effusions, it follows that in these cases the decrease in tension exceeds the decrease in volume. In other words, the tympanitic resonance obtained at the anterior superior portion of the affected side, in considerable pleuritic effusions, is due to a relatively greater decrease in the tension of the lung than in its volume.

This article is noticeable for the description of the author's treatment of empyema, which he has described at great length, with only a hint at the common modes of treatment. He has occasionally seen recovery followed by evacuation of the pus by means of the aspirator; therefore this operation is recommended for two or three times, if the pus withdrawn seems healthy; but if a cure does not result, or if the pus becomes unhealthy, the radical operation must be performed, to allow daily cleansing of the pleural sac.

The author's procedure is as follows: He dissects carefully down in the fourth or fifth intercostal space, somewhat external to the mammary line, until he reaches the pleura costalis, which he divides with great care. The incision made is something over two inches in length. Immediately the pleura is opened, two or three fingers are introduced into the wound to prevent too rapid escape of the pus. After the pus has escaped, the cavity is carefully washed out four or five times with distilled water. He then introduces a short silver cannula, having a circular silver plate, or flange, on its outer extremity, which rests against the chest wall, and must fit the surface accurately. The cannula must be large enough to admit two of Nélaton's medium-sized catheters; it is provided with a movable lid, which closes its orifice. A piece of *Lister's protective* is placed between the plate of the cannula and the surface of the wound. The cannula is then secured by plaster; it is covered with another piece of protective, and over this is placed a large compress of carbolized gauze, and the whole is secured by a bandage. An India rubber bag filled with ice is placed over the region of the wound, and absolute rest insisted on. The patient is compelled to lie very straight in bed to prevent curvature of the spine. Lister's carbolized spray must be used during the entire operation and dressing, and subsequently whenever the dressings are changed.

Twice every day the dressings are removed, and two of Nélaton's catheters introduced through the cannula to the lower part of the pleural cavity; through one of these water runs into the chest, and through the other it is pumped out until it comes away clear. The cannula must then be removed, carefully cleaned, and re-inserted, though this operation causes considerable pain after the wound has partially closed by granulations. The ice bag must be kept in position for two days, and distilled water used for that length of time to cleanse the cavity. Subsequently for several days a solution of common salt—gr. iiss. to ℥i.—is employed. Afterward compound tincture of iodine, diluted with twenty to fifty parts of water; or permanganate of potassium--gr. i. to ℥i. of water; or carbolic acid—gr. ii. to ℥i.—may be substituted.

He teaches that the cavity becomes obliterated by granulation and adhesions of the pleural surfaces. If during the treatment the sac ceases to diminish for a few days, the solution used for washing it out must be changed. As the result of all this care and subsequent tonic and climatic treatment, five out of his eleven cases have been completely cured; but we are not told how long they were under treatment. One was discharged with an incompletely healed thoracic fistula, one had been under treatment only two weeks at the time of writing, and four had died. He compares his cases with those of Moutard-Martin, who performed the radical operation in seventeen cases, but without the after treatment here recommended. Of these seventeen cases, five died and twelve are reported cured; but five of these were discharged with incompletely healed thoracic fistulæ. The other seven the author fears had some deformity of the chest which his five had not.

In estimating the value of this comparison, we must remember that the time these two sets of cases were under treatment is not given; and that the healing of thoracic fistulæ and recovery from deformities of the chest produced by empyema, is in many instances, only a matter of time.

The treatment sketched seems to us cumbersome and unnecessarily painful, while it is no more effectual than simpler methods. E. F. I.

An Introduction to Pathology and Morbid Anatomy. By *T. Henry Green, M.D.*, Lond., etc. Second American, from the third revised and enlarged English Edition. Illustrated by one hundred and eleven engravings on wood. Philadelphia: Henry C. Lea. 1876. pp. 316.

To any one at all acquainted with the earlier editions of this work little need be said, except that in the present the author has made such revision in the several chapters as was necessary to make the teaching of the book conform to the latest knowledge of the subjects under consideration; that much new matter has been added, and that the number of wood cuts has been considerably increased.

Those not acquainted with this text-book ought to be. We have always thought that for the average doctor this work was much more useful than some of the larger treatises. Into it is condensed much knowledge, to gain which elsewhere would require great labor and wide reading. For students and practitioners full of cares it is particularly valuable.

In this edition the general high character of the work is maintained; the new cuts are fully up to the standard of those used before, which were excellent; the execution of the work at the hands of the publisher is faultless.

We notice that of the thirty or more works bearing on pathology and morbid anatomy which the author has referred to, none are American, while only nine are British. We shall look for a different showing in the book on pathology, of our second centennial year.

A Contribution to the Medical History of our West African Campaigns. By Surgeon Major *A. A. Gore, M.D.*, late 34th Regiment; Sanitary Officer of the Staff of the Quartermaster General's Department during the Ashanti war of 1873. London: Bailliere, Tindall & Cox. 1876. pp. 219.

This little book does the author credit. As he says in his preface, he has endeavored "to collect together in systematic order the many interesting facts bearing upon the health and efficiency of the soldier in a malarial climate," which are scattered through the literature of the West African campaigns, and has added many personal notes and recollections.

The style of the writing is pleasant; and any one beginning an examination of it will dislike to lay it aside until it is finished.

There are chapters on the climate of the gold coast; on food supply, in its effect upon health; on beverages, alcoholic and others; on tobacco; on water supply; and on medico-chirurgical lessons, beside a number of others.

At the close of the chapter on "spirit ration," he says: "That it can be dispensed with in Africa by men of hardy frame and indomitable perseverance, under great exertion, has been proved by more than one great traveler; but that it does any harm, when taken in moderation at proper times and places, I do not believe."

Ophthalmic and Otic Memoranda. By *Dr. D. B. St. John Roosa*, Professor of Ophthalmology and Otology in the University of New York, Surgeon to the Manhattan Eye and Ear Hospital, and *Dr. Edward T. Ely*, Assistant to the Chair of Ophthalmology and Otology, University of New York, etc.

This very small work "aims to give a concise and correct outline of our present knowledge of ophthalmology and otology, and to serve as a kind of dictionary of these subjects." As such the work merits high commendation. The student who has impressed on his memory by careful study the meaning of every technical term and fully comprehends all the principles enunciated in the work, can follow intelligibly almost any course of clinical or didactic lectures on the eye or the ear.

The work is in duodecimo; price, $1.00.

